# Anti-Infective Antibody-Derived Peptides Active against Endogenous and Exogenous Fungi

**DOI:** 10.3390/microorganisms9010143

**Published:** 2021-01-10

**Authors:** Tecla Ciociola, Laura Giovati, Stefania Conti, Walter Magliani

**Affiliations:** Department of Medicine and Surgery, University of Parma, 43126 Parma, Italy; tecla.ciociola@unipr.it (T.C.); laura.giovati@unipr.it (L.G.); walter.magliani@unipr.it (W.M.)

**Keywords:** endogenous fungi, antifungal antibodies, antibody-derived antifungal peptides, antibody-derived anti-infective peptides, cryptides, antifungals, novel therapeutic approaches

## Abstract

Mycoses still represent relevant opportunistic infections worldwide, although overshadowed in recent years by other severe and more widespread infections. Moreover, deep-seated mycoses are often accompanied by unacceptably high mortality rates. Etiologic agents include endogenous components of the mycobiota, *Candida* and *Malassezia* species above all, and exogenous species, both yeasts and filamentous fungi. Old and new fungal pathogens are increasingly characterized by resistance to the existing antifungal agents, making imperative the search for effective and safe new therapeutics. Among the candidate molecules proposed in recent decades, synthetic peptides derived from the complementarity determining and constant regions of diverse antibodies (Abs), as well as the translated products of Ab-encoding genes, have proved of considerable interest. Their anti-infective activities, regardless of the specificity and isotype of the originating Ab, will be briefly presented and discussed in the light of their different mechanisms of action. Intriguing suggestions on the possible function of Abs after their half-life will be presented, following the recent detection, in human serum, of an antimicrobial Ab-derived peptide. Overall, Abs could represent a source of biologically active, highly flexible peptides, devoid of detectable toxicity, which can be easily synthesized and manipulated to be used, alone or in association with already available drugs, for new anti-infective strategies.

## 1. Introduction

In recent years, globalization and climate changes are favoring the emergence and rapid spread of old and new etiological agents, responsible for potentially life-threatening diseases, even in epidemic and pandemic form [1,2,3]. Moreover, the lack of effective therapeutic tools and vaccines and the growing spread of antimicrobial resistance may complicate the management of infectious diseases, putting the effectiveness of the available treatments at risk.

In this scenario, as recently outlined by Rodrigues and Nosanchuk, fungal diseases are mostly neglected [4]. Mycoses affecting the skin and its keratinized structures, hair and nails, are caused primarily by dermatophytes and *Malassezia*, other than *Candida* species. It is estimated that nearly one billion people are affected by skin mycoses; almost 20 percent of the world’s population [5,6]. Fungal infections of the mucous membranes are even more common, mostly in oral and genital tracts. Babies, denture wearers, individuals with hematological malignancies or treated with inhaled steroids, chemotherapy or radiotherapy, and transplanted patients are particularly prone to oral thrush. Oral and esophageal candidiasis are common in patients with HIV infection and AIDS. Genital candidiasis is of considerable epidemiological importance in women. This estrogen-dependent vulvovaginal infection, caused in most cases by *Candida albicans*, is probably the most frequent fungal infection all over the world. About 70 to 75% of women report at least one episode of candidal vulvovaginitis in their childbearing age, and 5 to 8% report recurrent vulvovaginal candidiasis (at least four episodes per year) [7].

A completely different scenario concerns deep-seated mycoses. Previously considered rare and of low impact on human health, since the 1980s, these mycoses are widely recognized as a serious clinical problem [8]. Since fungi are usually typical opportunistic pathogens, the incidence of deep-seated mycoses increased in the last decades in parallel with the increase in the number of individuals at risk. In fact, the ongoing medical progress allows an increasing critically ill patients to survive longer. Together with the elderly and premature babies, immunocompromised individuals, such as those affected by congenital or acquired immunodeficiencies, transplanted and oncological patients, are particularly prone to severe and often life-threatening fungal infections still associated with unacceptably high mortality rates [9,10,11,12]. Even in non-immunocompromised individuals, there may be predisposing healthcare-associated conditions, including the use of broad-spectrum antibiotics resulting in altered microbiota, heavy surgery, and long hospitalization [13,14,15].

The etiology of deep-seated mycoses is constantly evolving. In the 1980s, *Candida*, *Pneumocystis* and *Cryptococcus* species were the most common causative agents. More recently, infections caused by filamentous fungi, such as *Aspergillus* species and hyaline and black molds, have been increasing in number, while dimorphic fungi were and are quite common in endemic areas [16]. The exact reasons for these changes are not fully understood. While most of these fungal agents are exogenous, others, namely *Candida* and *Malassezia* species, are key components of the human mycobiota. By colonizing mucous membranes and/or the skin, they can take immediate advantage of local physiological/pathological changes of the host or medical interventions, such as antibacterial therapies and the use of medical devices, thus displaying their pathogenicity. Any variable that alters the commensal relationship with the host could be considered a risk factor for invasive infections, characterized by different clinical manifestations, depending on the patient’s clinical condition. At least eight species belonging to the genus *Malassezia* have been isolated from human skin. These lipophilic yeasts probably have a protective effect against colonization by more pathogenic species but can cause pityriasis versicolor and, indirectly, have also been associated with other dermatoses (folliculitis, atopic and seborrheic dermatitis, and psoriasis). Rarely, *Malassezia* species can also cause severe systemic infections in premature infants or immunocompromised patients due to the use of parenteral nutrition catheters [17] and have been associated with oncogenesis of pancreatic ductal adenocarcinoma [18]. Several *Candida* species normally live on the epithelial surfaces of healthy individuals as components of the normal human mycobiota and, under favorable conditions, can display unique properties, such as the production of virulence factors and biofilm, which contribute to their pathogenicity. At least 15 distinct species can be involved, as opportunistic endogenous pathogens, in human diseases and cause invasive, life-threatening infections in patients at risk. Yeasts of the genus *Candida* have been reported as the fourth most common cause of bloodstream infections, characterized by high mortality rates [19,20,21]. *C. albicans* remains the most frequent causative agent, but *Candida* non-*albicans* species now account for approximately 50% of infections, with a distribution dependent on geographic location and patient population. Among these, *C. glabrata*, *C. tropicalis*, *C. parapsilosis*, *C. krusei* and, more recently, *C. auris* have emerged as the prevalent pathogens. The worldwide trend of changing epidemiology is characterized by the emergence of strains and species that are resistant, refractory, or intrinsically less susceptible to available antifungal agents, with the consequence of an overall increase in mortality. Widespread use of antifungal drugs for prophylactic or therapeutic purposes, recently acquired virulence characteristics, and adaptation to the human body temperature have been suggested, among others, as determining factors for the emergence of fungal species [22,23,24,25,26]. Infections caused by other yeasts and filamentous fungi have been progressively reported, related to specific medical interventions and changes in the host, posing further significant challenges to human health [11,27]. Overall, invasive fungal infections have been associated with at least 1.5 million deaths worldwide each year [10,28]. The unavailability of effective, licensed antifungal vaccines represents another unsolved drawback [29].

## 2. Antifungal Agents

As fungi are eukaryotic organisms, many potential targets for therapeutic purposes are also present in human cells, with a significant risk of host toxicity. This has contributed to considerably slow down the introduction in the clinical use of antifungal agents, the last of which, the echinocandins, date back to 2002 [30]. Few classes of antifungal agents are currently used for the treatment of fungal infections [31,32,33]. Most of them recognize ergosterol in the fungal cell membrane as a specific target by inhibiting its biosynthesis, such as allylamines, azoles and morpholines, or interacting directly with the preformed molecule, such as polyenes.

The syntheses of β-(1,3)-D-glucans and chitin in the fungal cell wall are other specific targets for echinocandins, nikkomycins and polyoxins, respectively [30,34,35]. While the formers exhibit excellent safety profiles and low toxicity, the clinical application of the latter is compromised by their low in vivo activity. In particular, polyoxins are used globally as agricultural antibiotics. The synthesis of nucleic acids is inhibited by pyrimidine analogs, such as flucytosine (5-fluorocytosine, 5-FC). This pro-drug is taken up by fungal cells via a specific cytosine permease and is converted by a cytosine deaminase into 5-fluorouracil (5-FU) and other metabolites with fungistatic activity. Due to the frequent emergence of resistance, 5-FC is usually used in combination with polyenes or triazoles [36].

Other potential antifungal molecules include aureobasidin A [37], acting as inhibitors of sphingolipid biosynthesis, tavaborole [38], and sordarins [39,40], inhibitors of protein synthesis. However, these molecules suffer from some limitations, such as poor absorption in the gastrointestinal tract and short half-life, and their use is limited to hospital settings.

Overall, the available armamentarium is still limited to few antifungal agents, sometimes toxic and expensive, and increasingly associated with both intrinsic and acquired resistance [41,42,43]. As pointed out by Perfect [44], it is mandatory to improve the still unacceptably high mortality from fungal diseases (20–40%), by discovering less toxic and better fungicidal drugs to be used alone or in combination with existing ones. In this perspective, the antibodies (Abs) could represent an interesting and relevant source of new antifungal compounds.

## 3. Immune Responses against Fungi: Any Role for Antibodies?

There is widespread consensus that close collaboration between innate and cell-mediated immunity plays a critical role in defense against fungal diseases, as indirectly evidenced by the increased incidence of mycoses in patients with such immune deficiencies, including HIV infection, or undergoing immunosuppressive interventions. However, since the 1990s, experimental and clinical observations have contributed to assigning an important role to humoral immunity in antifungal protection [45]. The fungal cell is made up of a complex mosaic of antigenic epitopes against which a plethora of different Abs can be elicited. These can include protective, indifferent, and even infection-enhancing Abs, which can interfere with each other, dictating any possible immunoprotection. Isotype, specificity, and titer may also be crucial for protective activity [46]. Following a fungal infection or a vaccination, protective Abs may not be produced at effective titers. Protective epitopes may be poorly represented or hidden by more external immunodominant antigens. This certainly represents a further complication in the development of effective vaccines and is probably one of the reasons why no vaccines are currently in clinical use for any fungal pathogen [29].

Among the plethora of different Abs produced in the course of fungal infections, a family of fungicidal protective Abs aroused our particular interest. For some time, the research of our group had focused on a *Pichia anomala* (now *Wickerhamomyces anomalus*) killer toxin (PaKT), characterized by a broad-spectrum of antimicrobial activity in vitro. Unfortunately, PaKT is unstable under physiological conditions and, therefore, unable to exert its relevant antifungal activity in vivo. Anti-idiotypic Abs, mimicking the effect of PaKT, were raised in rabbits by idiotypic vaccination with a monoclonal Ab (mAb) able to neutralize the toxin. These affinity-purified Abs were able to directly kill *C. albicans* cells in vitro, thus acting as antibiotic-like molecules (“antibiobodies”). Using the same experimental approach (idiotypic vaccination), antibiobodies were raised in mice and rats and proved to be protective against intravenous or vaginal challenges with *C. albicans* cells, respectively. Antibiobodies could also be elicited in animals directly vaccinated with *C. albicans* cells bearing the PaKT receptor. Likewise, antibiobodies were detected in the vaginal fluid of women with vaginitis, suggesting a still undefined role in the anti-*Candida* Ab repertoire [47,48]. Based on these observations, rat monoclonal and recombinant single-chain fragment (scFv) antibiobodies were produced by idiotypic vaccination. As the previously described natural or experimentally elicited Abs, these antibiobodies were able to react with specific PaKT cell wall receptors on sensitive *C. albicans* cells and proved to be therapeutic in an experimental model of candidiasis. When tested under appropriate experimental conditions, antibiobodies exerted a broad-spectrum of in vitro and/or in vivo antimicrobial activity, including some *Candida* species, *Pneumocystis carinii* (now *P. jirovecii*) and *Aspergillus fumigatus*, bacteria, such as multidrug-resistant *Mycobacterium tuberculosis*, antibiotic-resistant Gram-positive cocci and oral streptococci, and protozoa, such as *Leishmania major*, *L. infantum* and *Acanthamoeba castellanii*. Such a broad-spectrum of antimicrobial activity could find a plausible explanation based on the nature of the receptor on the targeted microbial cells. β-glucans have been identified as PaKT receptors, while β-glucans or glucan-like molecules have been proposed as targets for antibiobodies [49]. Since β-glucans are absent in mammalian cells, new perspectives for transphyletic anti-infective control strategies have been envisaged, based on anti-β-glucan antibiobodies as novel broad-spectrum immunotherapeutics, including their expression in vivo from suitably engineered commensal bacteria [50], and β-glucan conjugates as new potential universal antifungal (antimicrobial) vaccines [51].

## 4. Anti-Infective Antibody-Derived Peptides

Among the decapeptides, which collectively reproduced the entire variable region of the recombinant scFv antibiobody, several showed fungicidal activity when tested against a *C. albicans* reference strain. The most active decapeptide was subjected to alanine scanning, thus obtaining the “killer peptide” (KP, sequence AKVTMTCSAS, Table 1), characterized by an implemented candidacidal activity. KP was the first Ab-derived peptide that proved to be active against numerous endogenous and exogenous fungi relevant to human disease, including *C. albicans* and other *Candida* species, *Cryptococcus neoformans*, *Malassezia* species and *Paracoccidioides brasiliensis*. The broad-spectrum of action has been further expanded to include bacteria, protozoa, such as *A. castellanii*, *Leishmania* species, *Toxoplasma gondii*, and, surprisingly, some relevant viruses, such as HIV-1, influenza A and herpes simplex [52,53,54].

**Figure 1:**
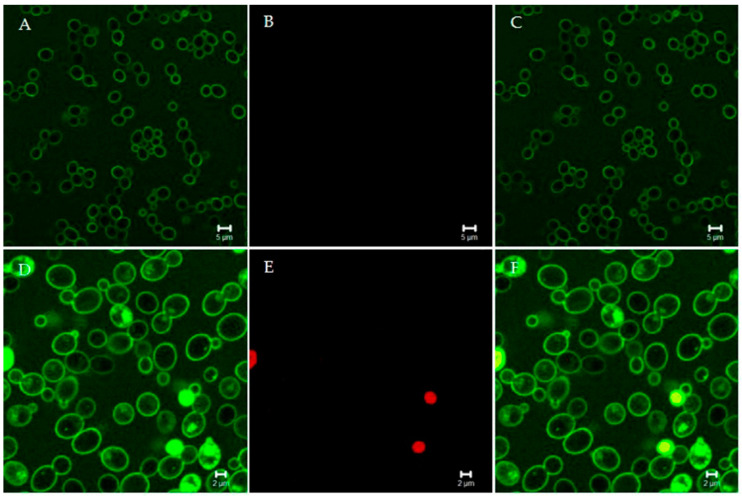
Confocal microscopy images of living *Candida albicans* cells treated with fluorescein isothiocyanate (FITC)-labeled “killer peptide” (KP). After 170 min of treatment, KP localized on the yeast cell surface (**A**). All yeast cells were viable, as assessed by propidium iodide exclusion (**B**). (**C**) merge of panels A and B. After 380 min of treatment, KP entered yeast cells (**D**). Some yeast cells were no longer viable, as assessed by propidium iodide internalization (**E**). (**F**) merge of panels D and E. Bar: 5 µm (panels A, B, and C); 2 µm (panels D, E, and F). The slow killing effect suggests a non-membranolytic mode of action.

Studies of the structure-function relationship of KP shed light on its wide spectrum of activity in vitro and its therapeutic effect in vivo. Dimeric KP has been shown to be the active form. Dimers are capable of spontaneously and reversibly self-assemble, leading to a conformation that resembles a physical hydrogel and explains the stability under physiological conditions. This process is favored by the alternation in the sequence of hydrophobic residues and hydrogen-bond donors and is strongly accelerated by 1,3-β-glucans, present on the *Candida* cell wall [59]. KP could interact with some other carbohydrates, and this, at least in part, may explain its broad-spectrum of activity against β-glucan- or β-glucan-like-bearing microorganisms. While KP does not show a pore-forming activity, as inferred by the slow kinetics of *C. albicans* killing (Figure 1), in-depth studies on antifungal mechanisms of action are still ongoing. On the other hand, the antiviral activity of KP is based on completely different and specific mechanisms for each viral agent. Downregulation of the CCR5 co-receptor and a physical block of the gp120-receptor interaction were suggested as possible mechanisms of KP activity against HIV-1. A marked reduction of influenza A proteins, especially M1 and HA, was observed in the late phase of viral multiplication, while a direct effect on viral particles was called into question as a major cause of the inhibition of herpes simplex virus. Furthermore, KP proved to interact with immune cells, mainly with dendritic and peripheral blood mononuclear cells, modulating their functions. Immunomodulatory activity possibly contributed to KP therapeutic effect in vivo and suggested a potential role of KP as an immunomodulator or vaccine adjuvant [60]. These characteristics, along with the lack of detectable toxicity in cultured cells and animal models, lead to KP being proposed as a lead compound for a new class of antimicrobial peptides, which can be easily synthesized or even expressed and produced in plants [52].

In the last decade, numerous other small peptides (10–30 amino acids) have been obtained from sequences of Abs available in the databases [61]. Peptides derived from Ab complementarity-determining region (CDR-peptides) have shown differential inhibitory activity in vitro, ex vivo and/or in vivo against *C. albicans*, but also against HIV-1 and even human melanoma cells, largely independent of the specificity of native Ab. Some of them could exert therapeutic effects by modulating immune cells, mainly macrophages, without possessing direct microbicidal activity. Selected peptides (Table 1), derived from the constant region of different Abs (Fc-peptides, N10K and T11F) or the translated products of Ab J and D heavy genes (Ab gene-peptides, L12P and L18R) [57] were fungicidal in vitro against various pathogenic yeasts (*C. albicans*, *C. glabrata*, *C. neoformans*, and *Malassezia furfur*), including strains resistant to conventional antifungals. Some of them proved to be therapeutic against experimental candidiasis in murine and *Galleria mellonella* models. Like other Ab-derived peptides, the Fc-peptide N10K exhibited immunoregulatory activity. All these observations led to the suggestion of Abs as an unlimited source of peptides with anti-infective activity [61].

The detection of CDR-peptides as a result of physiological Ab proteolysis in vivo would be extremely unlikely. On the other hand, the release in vivo of Fc-peptides from the constant region of numerous Abs, through the action of physiological proteases, may be plausible. The search for the previously described Fc-peptides in human sera by liquid chromatography coupled to high-resolution mass spectrometry (LC-HRMS) was, however, unsuccessful, at least within the detection limits of the system. Instead, a 40 amino acid phosphorylated peptide (K40H), derived from the Fc region of IgM Abs, was detected (Table 1). K40H displayed a fungicidal activity in vitro at micromolar concentration against yeast strains (*Candida*, *Cryptococcus* and *Malassezia* species) and a therapeutic effect against systemic candidiasis in *G. mellonella*, with no detectable toxicity on mammalian cells. Interestingly, K40H was also able to significantly inhibit HIV-1 replication in vitro and ex vivo [58].

The detection of K40H in human sera may lead to some intriguing considerations. As other large proteins, Abs could have formed during evolution through the assembly of ancestral genes encoding for peptides characterized by multiple intrinsic and nonspecific (antimicrobial, antiviral, immunomodulatory) biological functions. Moreover, the detection of biologically active Ab-derived peptides in human serum, following probable hydrolysis by physiological proteases, suggests that Abs may continue, through their fragments, to exert important biological functions beyond their half-life. These considerations help to blur the distinction between innate and adaptive immunity. Indeed, natural bioactive Ab fragments could represent a further tool to counteract microbial and viral infections [58].

These observations confirm what has been reported for several decades on the widespread generation of biologically active peptides (called cryptides) during the maturation or degradation processes of physiological proteins in serum, saliva, milk and many other sources, as recently reviewed by Iavarone et al. [62]. The described Ab-derived peptides could be fully included among the cryptides.

What do antifungal peptides derived from Abs or other physiological proteins have in common? First, it should be emphasized that not all the peptides showed biological activity, at least among those investigated in a necessarily limited number of models. As shown in Table 1, some common features seem to emerge among the considered peptides. The length between 10 and 40 amino acids, net positive charge, positive grand average of hydropathy (GRAVY) values, and alternation in the sequence of hydrophilic/hydrophobic amino acids appear to be features shared by many, though not by all, the described Ab-derived peptides. These properties are involved in the interaction with the negatively charged microbial surfaces and in antimicrobial activity. In fact, the replacement of positively charged residues with alanine proved to reduce the candidacidal activity [61].

Ab-derived peptides with antifungal activity often present a random coil conformation immediately after aqueous solution, as demonstrated by CD spectroscopy analysis. The Fc-peptide N10K, like KP, then undergoes conversion to β-sheet conformation, interacts with molecules superficially expressed on target yeast cells, afterward localizing inside and determining cellular alterations by the activation of various processes, including apoptosis, autophagy, and the production of reactive oxygen species. The FC-peptide T11F, on the other hand, can assume a polyproline II conformation. This peptide proved to bind to the yeast cell surface, then penetrate and cause leakage of cellular material [56].

**Figure 2:**
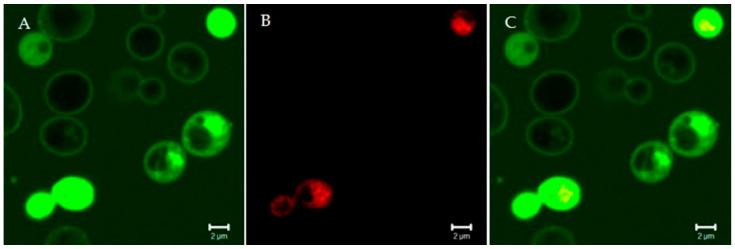
Confocal microscopy images of living *Candida albicans* cells treated for 15 min with the fluorescein isothiocyanate-labeled Ab gene-peptide L12P (**A**). The peptide was on the yeast cell surface and entered into yeast cells within a few minutes; empty vacuoles were seen. Some yeast cells were no longer viable, as assessed by propidium iodide internalization (**B**). (**C**) merge of panels A and B. Bar: 2 µm. The rapidity of the killing effect suggests a membrane-disrupting mode of action.

**Figure 3:**
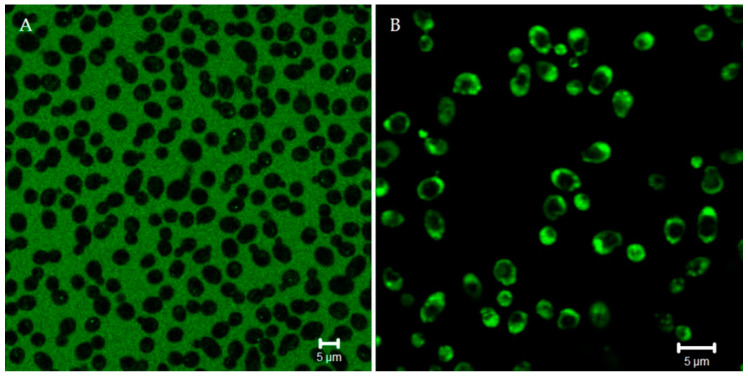
Confocal microscopy images of living *Candida albicans* cells pretreated with lucifer yellow (LY) (**A**). LY is a fluorescent molecule used as a quantitative detector of the cell membrane permeabilization. After the addition of unlabeled Ab gene-peptide (**B**), LY presence in yeast cells confirmed peptide-induced membrane permeabilization. Bar: 5 µm.

Other peptides, like the Ab-gene L12P—whose structure was not determined—proved to rapidly interact with yeast cells, causing membrane disruption and death (Figure 2 and Figure 3).

Although in some cases the precise antimicrobial mechanism is still undefined, Ab-derived peptides can be counted among the compounds of potential interest for the production of new antifungals and, more generally, antimicrobials, based on targets and mechanisms of action different from those of the drugs currently in use.

## 5. Conclusions

The need for new antifungal drugs, or more generally anti-infective drugs, becomes increasingly urgent, as widely emphasized above. Among the new molecules of potential interest, Ab-derived peptides may arouse particular interest for several reasons, including their transphyletic spectrum of activity and the lack of toxicity on mammalian cells and animal models. Like other peptides endowed with antimicrobial activity, Ab-derived peptides exhibit many advantages, along with some problematic characteristics.

The small size (10–40 amino acids) allows an easy and low-cost production by chemical synthesis and purification, as well as by recombinant technologies (engineered bacteria and yeasts, transgenic animals, and plants). Small peptides are highly flexible molecules, which can be easily manipulated by studying the relevance of individual amino acid residues and replacing them. This can lead to the implementation of antimicrobial activity, as in the case of KP and other Ab-derived peptides [55,56]. The insertion of unnatural amino acids and new functional groups as well as the conjugation to other molecules, including conventional drugs, cytotoxic compounds, and radionuclides, could improve anti-infective activity, stability, pharmacokinetics and pharmacodynamics of these molecules [63,64,65,66]. On the basis of the structure–activity relationships of the so far described Ab-derived peptides, a further opportunity to optimize and enhance their biological activity and stability could be the design and synthesis of consensus peptides. However, as a matter of fact, several Ab-derived peptides have already demonstrated sufficient stability and pharmacokinetic characteristics to be therapeutic against various experimental infections in animal models. Other relevant aspects relating to the use of these peptides as possible new anti-infective drugs still need to be investigated and clarified, such as the alleged lack of oral availability, the emergence of resistance and the mechanism of action. Regarding the latter, the studies on Ab-derived peptides seem to attest that at least some of them display, against different targets (microbial/viral), different mechanisms of action. The broad, transphyletic spectrum of activity of some Ab-derived peptides could represent a major drawback due to the impact that therapeutic treatments could have on the resident microbiota. This feature must be taken duly into account since narrow-spectrum antimicrobials are increasingly preferred.

Overall, peptides derived from Abs should be added to the long list of antimicrobial peptides under study in many research centers around the world [67] that could represent a promising exploitable alternative or complement to the currently available drugs.

## Figures and Tables

**Table 1:** Main features of the described antibody-derived peptides.

Peptide	Origin	Sequence	GRAVY Value ^1^	Net Charge	Structure	Antifungal Mechanism of Action	Ref.
**KP**	Variable region of a recombinant scFv-Ab	AKVTMTCSAS	0.530	1+	β-sheet	Interaction with cell wall molecules, intracellular targets	[52]
**N10K**	IgG Fc region	NQVSLTCLVK	0.610	1+	β-sheet	Apoptosis/ autophagy induction	[55]
**T11F**	IgM Fc region	TCRVDHRGLTF	−0.382	3+	PPII helix	Membrane binding and disruption	[56]
**L12P**	Translated product of IGLJ1 gene	LCLRNWDQGHRP	−1.292	2+	n.d. ^2^	Membrane disruption	[57]
**L18R**	Translated product of IGHJ2 gene	LLVLRSLGPWHPGHCLLR	0.467	4+	n.d.	Apoptosis induction	[57]
**K40H**	IgM Fc region	KSTKLTCLVTDLTTYDSVpTIpSWTRQNGEAVKTHTNISESH	−0.568	0	n.d.	Effect on actin dynamics	[58]

^1^ Grand average of hydropathy (GRAVY) calculated by the ExPASy tool ProtParam; ^2^ n.d., not determined.

## Data Availability

No new data were created or analyzed in this study. Data sharing is not applicable to this article.
